# The Impact of Organizational Commitment and Work Motivation on Retention Intention: Evidence from Long-Term Care Institution Caregivers Based on Expectancy Theory

**DOI:** 10.3390/healthcare13222832

**Published:** 2025-11-08

**Authors:** Szu-Han Yeh, Kuo-Chung Huang

**Affiliations:** Department of Business Administration, Nanhua University, Chiayi County 622301, Taiwan; kchuang@nhu.edu.tw

**Keywords:** long-term care institution, retention intention, expectancy theory, work values, work engagement

## Abstract

**Background/Objectives**: With the increase in the aging population and disabilities, long-term care facilities in Taiwan are experiencing workforce shortages that threaten sustainability. This study uses expectancy theory to examine the influence of organizational commitment and work motivation on retention intention, with work values as mediators of the relationships and work engagement as a moderator. **Methods**: Data were collected from 532 care attendants using purposive sampling. After excluding invalid responses, structural equation modeling analysis was conducted on 501 responses. **Results**: The results revealed that organizational commitment and work motivation do not directly predict retention intention but have positive indirect effects on retention intention through intrinsic and extrinsic work values, with extrinsic work values exerting more substantial indirect effects. The results also showed that work engagement positively and significantly strengthened the relationships between the antecedents to work values. **Conclusions**: This suggests that retention is more impacted by positively recognizing work values or caring for residents than by commitment and motivation. Theoretically, this study extends expectancy theory to long-term human resource management in care facilities. Practically, this reveals a need for high-performance work systems shaped by reward systems to improve engagement, stabilize the workforce, and decrease turnover.

## 1. Introduction

With the global trend of population aging and the rising proportion of individuals with disabilities, long-term care (LTC) service institutions have become a critical issue in public policy. As of 2024, there were 3619 LTC institutions across Taiwan, with the industry scale conservatively estimated at over NT$660 billion in 2022, and it continues to expand with the aging population [1]. Although the government has actively promoted caregiver training, by the end of April 2022, there were approximately 90,000 caregivers nationwide, with home care representing the largest share (48,467 caregivers). Nevertheless, this number remains insufficient to meet demand [2], resulting in generally high workloads for personnel.

The intersection of workforce shortages and high turnover rates poses challenges to the sustainable operation of LTC institutions. Tazi et al. [3] reported that the voluntary turnover rate in LTC institutions reached 45.41%. Voluntary turnover not only increases recruitment and training costs but also leads to the loss of knowledge and social capital [4], thereby undermining service quality [5]. Turnover may further trigger chain reactions [6], and for caregivers—whose roles require long-term development of professional and interactional skills—such losses are particularly detrimental.

Organizational commitment serves as the foundation for employees to demonstrate loyalty and sustained involvement, which supports capacity building and enhances service quality [7]. Work motivation is a critical factor influencing employees’ retention intention. Defined by Pinder [8] as the psychological force driving employees to engage in work, motivation is regarded as a key determinant of organizational success [9]. Based on self-determination theory (SDT), motivation is not only associated with performance [10] but also positively related to creativity and productivity [11].

This study also highlights the importance of work values and work engagement. As a central concept in positive psychology [12], work engagement fosters motivation and improves performance. According to Expectancy Theory, an individual’s behavioral motivation depends on the cognitive evaluation of the relationship between effort and outcomes [13], emphasizing three key elements: expectancy, instrumentality, and valence. Among them, valence reflects the individual’s perceived importance of the outcome. When employees believe that their efforts can lead to valuable results, their work engagement and intention to remain in the organization increase [14,15]. Therefore, Expectancy Theory serves as an essential theoretical foundation for understanding how care attendants form retention intentions through the perception of work value. Prior research has demonstrated its mediating role in shaping outcomes [16,17,18]. Employees with high work engagement are more likely to exhibit professionalism and stability, thereby strengthening trust and enhancing organizational sustainability and competitiveness.

Although existing studies have explored the effects of organizational commitment, work motivation, and work values on employee behavior [19,20], few empirical studies within the LTC sector have examined the integrated effects of these constructs on retention intention while incorporating work engagement as a moderating factor. Furthermore, gaps remain in the literature regarding how expectancy theory can explain caregivers’ perceptions of work values under varying levels of work engagement, and how such perceptions further influence retention intention. Against this backdrop, this study investigates the effects of organizational commitment, work motivation, and work values on caregivers’ retention intention in LTC institutions, while verifying the mediating role of work values and the moderating role of work engagement. The findings aim to provide concrete managerial implications for sustainable human resource management in the LTC sector.

This study focuses on employees in LTC service institutions. First, based on the perspective of expectancy theory and SDT, it explores the relationships among organizational commitment, work motivation, work values, and retention intention, with the goal of establishing a more comprehensive analytical model applicable to management practices, thereby assisting the LTC sector in achieving sustainable human resource development. Second, it examines the mediating role of work values—both intrinsic and extrinsic—in the process by which organizational commitment and work motivation influence retention intention. Finally, it investigates how varying levels of work engagement shape employees’ perceptions of work values, in order to clarify how work engagement functions as an external factor influencing employees’ work evaluation and retention intention. In turn, the study provides empirical evidence with practical relevance for stabilizing the workforce and strengthening talent retention strategies in LTC institutions.

## 2. Literature Review

### 2.1. Theoretical Foundation

#### 2.1.1. Expectancy Theory

Expectancy theory, proposed by Vroom [13], explains that individual behavior depends on the cognition and evaluation of outcomes [14]. The theory is built on the foundation of rational choice in human behavior. It emphasizes that individuals decide whether to take action based on their judgment of the relationships among effort, performance, and outcomes [21]. Expectancy theory posits that motivation is driven by expectancy, instrumentality, and valence. The combination of these factors determines the intensity and direction of individual action [13,15]. When individuals perceive a positive relationship between effort and performance, a strong linkage between performance and rewards, and assign high value to rewards, stronger work motivation can be stimulated.

The perspective of expectancy theory helps understand employees’ retention, particularly in the LTC industry, where sustainable human resource management and a stable professional workforce are essential for ensuring service quality and continuity [15]. When organizations provide precise performance evaluation and feedback mechanisms and closely align performance with compensation, benefits, training, and promotion opportunities, employees can develop positive perceptions regarding expectancy and instrumentality [22]. Moreover, when rewards design corresponds with employees’ career planning, work values, and life goals, the valence dimension can also be enhanced [16]. Through such cognitive processes, employees choose to remain in their positions and contribute professional expertise and experience to the organization over the long term. Strengthening this motivational mechanism helps shape a sustainable service team, enabling the LTC industry to maintain stable, high-quality care services amid an aging population and increasing service demands.

#### 2.1.2. Self-Determination Theory

SDT [23] is an important theoretical framework for explaining human behavioral motivation. Its core lies in distinguishing between autonomous motivation and controlled motivation. Autonomous motivation refers to actions that are self-determined based on intrinsic interest or personal values, while controlled motivation arises from external rewards, pressures, or social expectations [24]. SDT posits that motivation exists on a continuum ranging from autonomous to controlled forms [25], encompassing entirely self-directed intrinsic motivation and externally regulated motivation. In contrast, amotivation reflects a lack of behavioral intention and sense of purpose [26].

This theory emphasizes three fundamental psychological needs: autonomy, competence, and relatedness. When these needs are fulfilled, individuals experience a sense of freedom and value congruence in their actions, thereby enhancing intrinsic motivation. Conversely, excessive external regulations and conditional rewards weaken intrinsic drive [10,23]. Furthermore, external motivation can be transformed into more autonomous forms through the processes of internalization and integration, promoting more stable and positive behavioral outcomes [24].

In the context of LTC institutions, care attendants face high emotional and physical demands, and the quality of their motivation directly affects their intention to remain. According to Deci et al. [10], when care attendants’ work is driven by a sense of service and self-value, they demonstrate greater job satisfaction and persistence. Therefore, when LTC institutions meet attendants’ psychological needs and foster a supportive organizational climate, employees are more likely to exhibit work motivation, develop proper work values, and consequently strengthen their retention intention.

#### 2.1.3. Theoretical Foundation of the Study

Based on the above literature, Expectancy Theory elaborates the rational decision-making process of nursing attendants under the organisational systems and rewarding situations presented to them [22], while SDT adds to it the explanation of the internal process of the psychological need fulfilment and motivational quality [25]. The two theories are interrelated in theory. The first deals with how outside incentives induce commitment and involvement, while the latter elucidates how motivation maintains continuity and retention. This theoretical linkage enables a deeper understanding of the mechanism underlying the intention to retain nursing attendants. It establishes a theoretical foundation for sustainable human resource development and motivational management in LTC institutions.

Thus, it is found that the theoretical foundation of the present study rests on expectancy theory [13], and SDT is added to it to incorporate both the rational-cognitive and the psychological-need approaches to explaining the long-term retention intention of nursing attendants in LTC institutions. Expectancy theory is used to elucidate the mediating role played by commitment to the organization within the research model. At the same time, SDT is invoked to explain the effect of work motivation in the first instance. The combination of the two approaches goes some way toward overcoming the limitations of relying on only one theoretical construct and provides a comprehensive mechanism of explanation, illustrating the joint influence of external incentives and intrinsic motivation.

### 2.2. Hypotheses Development

#### 2.2.1. Organizational Commitment

Organizational commitment is regarded as employees’ emotional attachment, identification, and involvement with the organization, manifested in their willingness to maintain membership and strive toward organizational goals [27]. It encompasses three dimensions—affective, normative, and continuance [28]. Affective commitment arises from a sense of belonging and emotional connection, normative commitment is based on responsibility and loyalty, and continuance commitment derives from considering the costs of leaving [29]. This psychological state closely links employees with the organization [30]. It is particularly significant in LTC services, directly related to stability and sustainability. Research by Azmy et al. [7] and Redondo et al. [31] has shown that organizational commitment can enhance job satisfaction and workplace well-being while reducing turnover intention. Within LTC institutions, highly committed employees are more willing to support the organizational mission and sustainable development continuously, prioritizing service quality and reputation over personal interests [4]. Such psychological bonding helps employees maintain a positive attitude and stable performance despite caregiving’s high emotional and physical demands, thereby reducing workforce loss and sustaining competitiveness.

However, existing research has primarily focused on the direct effects of organizational commitment on performance and turnover intention [32,33], while its indirect mechanisms remain underexplored. According to expectancy theory, when employees perceive expectancy, instrumentality, and valence, they are likelier to engage and remain in their positions [12,14]. This suggests that organizational commitment may indirectly influence retention intention through work values and work engagement. However, few studies have examined this psychological process in the LTC context [34,35], particularly how affective commitment strengthens the recognition of work values, enhancing sustained engagement and retention motivation. Within the sustainable human resource management framework, how organizational commitment promotes work engagement and stabilizes workforce structures remains an insufficiently addressed issue in the current literature, constituting an important theoretical gap that this study seeks to address.

#### 2.2.2. Work Motivation

Work motivation drives individuals to initiate, direct, and sustain goal-oriented behaviors, with its process encompassing direction, intensity, and persistence [11]. According to SDT, motivation can be classified into autonomous and controlled types, where intrinsic motivation refers to engagement in an activity for the inherent satisfaction it provides [36]. Autonomous motivation also involves the identification and internalization of the value of an activity; when individuals perceive voluntariness and self-endorsement, they demonstrate a high degree of autonomy [10,37]. In LTC services, intrinsic motivation reflects interest and involvement in caregiving tasks. In contrast, autonomous motivation manifests as freely engaging in care based on enjoyment and recognition of its significance.

Work motivation is shaped by both internal and external factors [38]. Intrinsic factors are often associated with the pursuit of high standards of achievement [39]. For LTC employees, perceiving their work as interesting, challenging, and socially meaningful is linked to higher satisfaction and performance, whereas extrinsic motivation is primarily driven by monetary compensation or social recognition [40]. Overall, motivation represents a behavioral tendency formed in response to internal and external stimuli and is closely tied to the drive to achieve care-related goals [40].

High levels of motivation enhance intrinsic satisfaction and facilitate a conscious response to needs [41]. Since LTC work requires substantial emotional labor and interpersonal interaction, autonomous motivation maintains employees’ physical and psychological well-being [9]. Intrinsic motivation is positively associated with creativity, effort, and performance [36] and also influences how employees cope with stress. Trépanier et al. [42] found that employees with low autonomous motivation are more prone to psychological distress in high-demand environments. In contrast, those with high autonomous motivation can more effectively regulate stress. In LTC practice, this directly affects service quality, resident safety, and the continuity of care relationships. The well-being and stable engagement derived from autonomous motivation help reduce turnover, preserve knowledge accumulation, and ensure service sustainability [41]. However, how work motivation further influences the retention intention of LTC employees remains an issue that requires deeper exploration.

#### 2.2.3. Retention Intention

Retention intention is an important psychological indicator for assessing whether employees are willing to remain engaged in the long term. It refers to individuals’ tendency to stay in a specific position or organization [43] and is regarded as a core construct for addressing workforce shortages. In nursing and LTC contexts, retention intention is often measured by directly asking whether employees intend to remain in their positions and is considered the reverse construct of turnover intention [44]. Given the long-term and stable demand for LTC, maintaining retention intention is essential for service quality and industry sustainability. Its influencing factors include compensation, flexible working hours, job stability, interpersonal relationships, leadership style, and organizational trust [45]. Supportive systems and career development can increase satisfaction and strengthen retention, while significant gaps between conditions and expectations can lead to higher turnover rates [46]. Thus, creating an organizational environment that fosters satisfaction and a sense of belonging contributes to employee retention and sustainable caregiving. However, prior research has primarily focused on external conditions, with limited exploration of the deeper relationship between affective organizational commitment and retention. Literature has shown that organizational commitment is a significant predictor of retention [47], but under the high-pressure environment of LTC, how organizations shape culture, provide support mechanisms, and develop career planning to foster enduring retention intention remains an underexplored issue.

From the perspective of expectancy theory, whether LTC employees choose to remain depends on their cognition of the relationship between behavior and outcomes. The theory posits that the strength of motivation arises from three key beliefs: expectancy, instrumentality, and valence [15]. When individuals strongly value the perceived rewards, valence is positive, and they are more likely to invest effort in pursuing outcomes [15]. This theory emphasizes rational choices in pursuing favorable outcomes, highlighting the trade-offs between benefits and costs. In the LTC context, the cognitive process of “effort–performance–reward” directly influences employees’ remaining decisions, thereby sustaining service continuity and care sustainability.

Organizational commitment represents employees’ emotional attachment to and identification with the organization, motivating their willingness to contribute and maintain membership [48]. Within the framework of expectancy theory, employees with higher affective commitment are more likely to believe that their efforts will be recognized and transformed into performance acknowledgment, thereby enhancing expectancy and valence and strengthening retention intention. Empirical studies further show that organizational commitment significantly predicts retention and turnover intentions [47]. Employees with high commitment are less likely to be absent or leave [49] and demonstrate stable retention tendencies across organizational contexts [50]. Moreover, organizations that provide fair compensation, flexible working hours, safe workplaces, supportive leadership, and career development opportunities can effectively enhance employees’ work motivation and strengthen retention intentions [51]. Conversely, unmet conditions or barriers to employees’ needs increase turnover tendencies [46]. Therefore, this study applies expectancy theory to explain the retention intention of LTC employees and proposes the following hypothesis:

**H1.**  *Organizational commitment positively influences retention intention.*

**H2.**  *Work motivation positively influences retention intention.*

#### 2.2.4. Work Values

Work values serve as a core indicator for evaluating the meaning and importance of work among LTC employees, reflecting their preferences and beliefs regarding work behavior and states of being. Rokeach [52] defined values as enduring beliefs that a particular mode of conduct or state of existence is preferable to its opposite [53]. Work values are also regarded as important standards through which individuals judge the significance of their work [54].

Work values are generally divided into intrinsic and extrinsic dimensions [55,56]. Extrinsic values emphasize salary, stability, and security, whereas intrinsic values emphasize autonomy, challenge, and growth [53]. In the LTC context, intrinsic values are reflected in a sense of mission, autonomous decision-making, and professional development, while extrinsic values involve salary, stability, and benefits. Both dimensions influence employees’ organizational commitment and retention intention. Moreover, work values may shift with environmental changes. Cao [55] noted that extrinsic values gain importance during economic instability. Work values are also closely associated with psychological need satisfaction, work engagement, and life satisfaction [44]. Given the high labor and emotional demands of the LTC industry, balancing intrinsic and extrinsic values affects employees’ retention intentions and relates to the sustainability of the sector’s workforce development.

Organizational commitment reflects employees’ emotional attachment, identification, and organizational involvement. When employees perceive that the organization consistently invests in job content, resource support, and career development, their confidence in work outcomes and recognition of outcome value are enhanced [57]. Based on expectancy theory, at the intrinsic work values level, strong organizational commitment can encourage employees in LTC services to perceive autonomy, challenges, and opportunities for growth, reinforcing their sense of mission and meaningfulness at work [53]. At the same time, organizational commitment is also closely related to extrinsic work values; when employees perceive organizational support in terms of salary, job stability, and welfare systems, their valuation of extrinsic rewards is strengthened [58]. Therefore, the following hypothesis is proposed:

**H3.**  *Organizational commitment positively influences intrinsic work values.*

**H4.**  *Organizational commitment positively influences extrinsic work values.*

Expectancy theory posits that employees form motivation based on the belief that effort can lead to good performance, performance can result in desired outcomes, and such outcomes are personally valuable [22]. In the context of LTC, characterized by high interpersonal interaction and emotional labor, enhanced work motivation can strengthen employees’ pursuit of intrinsic values, such as achieving self-fulfillment, improving skills, and gaining professional autonomy through caregiving [58]. Furthermore, high levels of work motivation also increase employees’ emphasis on realizing extrinsic rewards, including fair compensation, job stability, and favorable working conditions [53]. Therefore, the following hypothesis is proposed:

**H5.**  *Work motivation positively influences intrinsic work values.*

**H6.**  *Work motivation positively influences extrinsic work values.*

The influence of intrinsic and extrinsic work values on employees’ retention intention is particularly critical in LTC. Intrinsic work values enable employees to derive meaning and fulfillment from their work; this psychological resource helps protect well-being and sustain service enthusiasm when facing high pressure and challenges [59]. Extrinsic work values are directly related to employees’ evaluation of job security and quality of life; when external rewards align with employees’ expectations and needs, retention intention significantly increases [60]. Therefore, the following hypothesis is proposed:

**H7.**  *Intrinsic work values positively influence retention intention.*

**H8.**  *Extrinsic work values positively influence retention intention.*

#### 2.2.5. Work Values as Mediating Variable

Based on the above discussion, this study argues that intrinsic and extrinsic work values may mediate between organizational commitment and retention intention. High organizational commitment can enhance employees’ intrinsic value perceptions, strengthen their emotional connection and sense of mission toward work, and promote retention intention [53]. At the same time, organizational commitment can also reinforce the fulfillment of extrinsic values, increasing employees’ trust in job stability and rewards, enhancing retention intention [58]. Therefore, the following hypothesis is proposed:

**H9.**  *Intrinsic work values mediate the relationship between organizational commitment and retention intention.*

**H10.**  *Extrinsic work values mediate the relationship between organizational commitment and retention intention.*

Similarly, work motivation may influence retention intention through intrinsic and extrinsic work values. High work motivation can strengthen employees’ pursuit of intrinsic values in LTC services, such as challenge, self-actualization, and professional growth, thereby enhancing their long-term commitment to the organization and retention intention [59]. At the same time, greater work motivation may also increase employees’ evaluation of the importance of extrinsic rewards; when these needs are met, their decision to remain is further reinforced [53]. Therefore, the following hypothesis is proposed:

**H11.**  *Intrinsic work values mediate the relationship between work motivation and retention intention.*

**H12.**  *Extrinsic work values mediate the relationship between work motivation and retention intention.*

#### 2.2.6. Work Engagement

Work engagement is a positive, fulfilling, and work-related psychological state that includes vigor, dedication, and absorption [61]. Kahn [62] described it as the process through which employees invest themselves physically, cognitively, and emotionally in their roles, while subsequent studies have highlighted its independence and multidimensional characteristics [63]. In the LTC context, work engagement involves not only task execution but also deep emotional and professional involvement, directly influencing care quality and the well-being of service recipients. It also serves as an essential foundation for sustaining the workforce.

Employees with high engagement demonstrate enthusiasm and energy, which can be transformed into proactive behaviors and high-quality services [61]. In LTC settings, they can endure high emotional labor, demonstrate empathy and professional judgment, and thus enhance service quality while reducing turnover. This contributes significantly to industry stability and sustainable development. However, how LTC employees maintain engagement in high-pressure and long-hour work environments has yet to be systematically examined. With increasing aging populations and rising demand, a lack of engagement may lead to turnover and declining service quality, threatening industry sustainability. Moreover, few studies have simultaneously investigated the interaction among organizational support, job characteristics, and intrinsic motivation in shaping engagement. This study, therefore, further explores the critical role of work engagement in LTC services.

According to expectancy theory, the extent to which employees engage in work depends on their cognition of the relationships among effort, performance, and outcomes and their evaluation of outcome value [14]. When employees perceive that organizational commitment or work motivation can bring about work outcomes aligned with their needs and expectations, motivational strength increases, shaping positive perceptions of work values [64]. Organizational commitment reflects employees’ emotional attachment, identification, and organizational involvement [27]. Intrinsic work values emphasize autonomy, growth, and meaningfulness, whereas extrinsic work values focus on salary, stability, and security [53]. This study argues that when employees possess high work engagement, the influence of organizational commitment on work values is likely to be amplified in the LTC context. Highly engaged employees can transform organizational support and resources into self-growth and professional achievement, thereby strengthening the positive relationship between organizational commitment and intrinsic work values. Similarly, when employees are highly engaged, organizational provisions such as compensation, security, and resources are more likely to be perceived as important and attractive, intensifying the influence of organizational commitment on extrinsic work values. Therefore, the following hypothesis is proposed:

**H13.**  *The positive relationship between organizational commitment and intrinsic work values is moderated by work engagement, such that the relationship is stronger when work engagement is high.*

**H14.**  *The positive relationship between organizational commitment and extrinsic work values is moderated by work engagement, such that the relationship is stronger when work engagement is high.*

**Figure 1:**
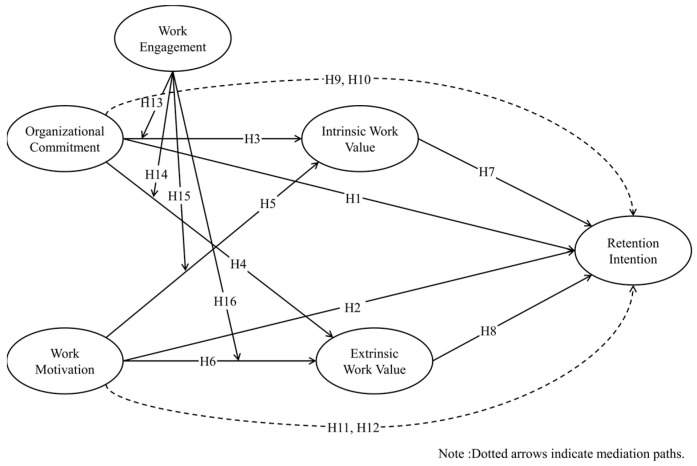
Conceptual model.

Expectancy theory also stresses that motivation develops as a function of employees’ perceptions that effort leads to performance, and performance results in outcomes perceived as valuable. Work motivation consists of intrinsic and extrinsic components; the former is derived from the participant’s interest in the work and the challenge of the work itself, while the latter includes external motivation such as rewards, recognition, and promotion [65]. If employees are highly engaged or exhibit a positive psychological state, this phenomenon may enhance the relationship between motivation and work values. For an employee with high intrinsic motivation, high engagement leads to more deliberation and focus on work outcomes such as achievement, self-actualization, and professional development, which enriches the positive relationships between work motivation and intrinsic work values. Conversely, for an employee with high extrinsic motivation, high engagement leads to increased awareness and responsiveness to the favorability of external outcomes such as compensation, stability, and benefits, nurturing the impact of work motivation of extrinsic work values. The hypothesis, then, will be (Figure 1):

**H15.**  *The positive relationship between work motivation and intrinsic work values is moderated by work engagement, such that the relationship is stronger when work engagement is high.*

**H16.**  *The positive relationship between work motivation and extrinsic work values is moderated by work engagement, such that the relationship is stronger when work engagement is high.*

## 3. Research Methodology

### 3.1. Sample and Procedure

This study investigates the retention intention of LTC service employees, with the research sample drawn from individuals currently employed in LTC services in Taiwan. A purposive sampling method was employed, which falls under the category of non-random sampling. This method enables the data to better reflect the characteristics of the people being studied and to increase the representativeness of the results. However, the process of sampling is subject to the researcher’s subjectivity, e.g., LTC institutions that are more willing to participate will be selected, which tends to yield more favourable outcomes. Although purposive sampling has these limitations, the author considers that the concentration and relevance of the data are more important to the validity of the results. Hence, purposive sampling was employed in this research.

To examine the proposed research model, data were obtained from a questionnaire survey administered between March and June 2025 to 600 LTC employees across institutions in Taiwan. Questionnaires were distributed on-site and on paper, with researchers providing in-person instructions. Completed questionnaires were immediately checked for completeness to ensure data validity. Following the sample size estimation rule, which requires at least 10 times the number of measurement items (46 in total), this study expected to collect a minimum of 460 valid responses. A total of 532 questionnaires were collected, and after excluding incomplete or invalid responses, 501 valid samples were obtained.

Regarding demographic characteristics, most respondents were female (393, 78.4%), while 108 were male (21.6%). In terms of age distribution, the largest group was aged 51–60 years (160, 31.9%), followed by 61–65 years (89, 17.8%) and 41–50 years (94, 18.8%). Concerning years of service, 180 participants (35.9%) had 5–15 years of tenure, 132 participants (26.3%) had 2–5 years, and 57 participants (11.4%) had 1–2 years of experience. As for educational attainment, the largest group held a high school or vocational school degree 243 participants (48.5%), followed by junior high school or below for 147 participants (29.3%), associate degree for 59 participants (11.8%), and university or above for 52 participants (10.4%). Detailed demographic data are presented in Table 1.

### 3.2. Instrument

Since the participants of this study were LTC service workers in Taiwan, the back-translation method proposed by Brislin [66] was adopted. Two bilingual experts proficient in both Chinese and English were invited to ensure that the semantic meaning was not altered during the translation process from English to Chinese, thereby confirming the accuracy of the Chinese items used in this study. The questionnaire of this study was designed with five main sections measuring organizational commitment, work motivation, work values, retention intention, and work engagement, in addition to demographic information of respondents. To ensure reliability and validity, the items of each construct were adapted from prior empirical studies and modified appropriately to fit the research context. All items were measured using a five-point Likert scale, where 1 represented “strongly disagree” and 5 represented “strongly agree,” in order to quantify the respondents’ level of agreement.

The first section, organizational commitment, included 9 items adapted from Meyer et al. [67] to measure respondents’ affective and continuance commitment toward their organizations. The second section, work motivation, consisted of 10 items revised from Kuvaas et al. [68] to examine the sources of respondents’ motivation to engage in LTC work. The third section, work values, comprised 10 items across two dimensions: intrinsic and extrinsic values (5 items each), adapted from Liang [69], to assess respondents’ perceptions and preferences regarding work values. The fourth section, retention intention, included 7 items based on Chen & Chen [70], designed to evaluate respondents’ willingness to remain in their current positions. The fifth section, work engagement, included 9 items adopted from Labrague & Obeidat [71], measuring respondents’ level of involvement and enthusiasm in their work. The complete scale can be found in Appendix A.

### 3.3. Ethical Considerations

This research was approved and cleared by the Quantitative Analysis and Research Association (No. 1140211001).

### 3.4. Data Analysis

The data collected in this study were analyzed using structural equation modeling (SEM), with AMOS statistical software version 24 employed as the analytical tool to verify the relationships among the research variables. This study selected AMOS as the data analysis tool primarily because the research model is confirmatory in nature. AMOS is a program designed for covariance-based structural equation modeling (CB-SEM), a powerful tool for assessing the overall fit of the model and the significance of each path relationship. Additionally, AMOS offers a user-friendly graphical interface and comprehensive model modification indices, enabling accurate analysis and interpretation of results. The data analysis process first conducted confirmatory factor analysis (CFA) to examine the reliability and validity of each construct scale. Subsequently, path analysis based on SEM was performed to evaluate the direct effects of the research hypotheses. The bootstrap method proposed by Hayes [72] was adopted to test mediation effects, with 5000 resampling iterations conducted to estimate the indirect relationships of mediation in the structural paths. In testing moderation effects, the method suggested by Ping [73] was applied to analyze the moderating role of work engagement on the relationships between organizational commitment, work motivation, and work values. The model also included interaction terms and was subjected to significance testing to ensure the statistical explanatory power of the moderation effects and the robustness of the study’s conclusions.

### 3.5. Common Method Bias

Since this study adopted a self-reported questionnaire for data collection, attention was given to the potential issue of common method bias (CMB) [74]. In the preventive stage, following the recommendations of Podsakoff et al. [75], anonymity of respondents was ensured, and measurement items were pretested and adapted from established studies to minimize the influence of CMB. Furthermore, items from different constructs were randomly mixed in the questionnaire, and the research purpose and variable names were concealed to enhance the authenticity of responses [76]. In the post hoc examination, Harman’s single-factor test was first conducted to assess the degree of CMB. The unrotated factor analysis results indicated that the first principal component explained only 42% of the variance, which did not exceed the 50% threshold.

## 4. Results

### 4.1. Measurement Model

Before conducting measurement model analysis, the sample data were first examined for univariate normality. The statistical results indicated that the absolute values of skewness ranged from 0.042 to 0.925, while the absolute values of kurtosis ranged from 0.041 to 1.367. All values fell within the recommended thresholds proposed by Kline [77], which suggest that absolute skewness should be less than 2 and absolute kurtosis should be less than 7. These results demonstrate that the data met the requirement of univariate normality and were suitable for subsequent SEM analysis. A CFA was then performed to assess the convergent validity of the measurement model. According to Hair et al. [78], convergent validity is established when all standardized factor loadings exceed 0.6, composite reliability (CR) is greater than 0.7, and the average variance extracted (AVE) is greater than 0.5. As shown in Table 2, the analysis results revealed that the factor loadings, CR, and AVE of all constructs met the recommended criteria of Hair et al. [78], indicating that the latent variables in this study possessed good measurement consistency and convergent validity.

Second, Hair et al. [78] indicated that discriminant validity is established when the absolute value of the Pearson correlation coefficient between constructs is smaller than the square root of each construct’s AVE. As shown in Table 3, the absolute values of all Pearson correlation coefficients (lower triangle) were less than the square root of AVE values presented on the diagonal, confirming good discriminant validity of the measurement scales in this study. In addition, Kline [77] suggested that constructs can be considered distinct if the absolute value of their Pearson correlation coefficient is less than 0.850. According to the results of this study, the correlation coefficients among constructs ranged from 0.096 to 0.635. Since extrinsic work values and intrinsic work values are subdimensions of work values, their correlation coefficient was relatively higher at 0.874, yet still well below the threshold. These findings further support that the constructs in this study demonstrate good discriminant validity.

### 4.2. Structural Model

The model fit analysis results showed that the Bollen-Stine χ^2^ was 825.499 with 585 degrees of freedom, yielding a χ^2^/DF ratio of 1.411, which falls within the recommended range. The overall model fit indices also indicated good performance: GFI = 0.943, AGFI = 0.934, RMSEA = 0.029, SRMR = 0.070, TLI = 0.981, CFI = 0.983, IFI = 0.983, Hoelter’s N = 355.419, and Gamma hat = 0.988. In sum, all indices met or were close to the ideal standards recommended by Bagozzi & Yi [79] and West et al. [80], demonstrating that the structural model exhibited good fit and adequately represented the structural relationships of the study population.

**Figure 2:**
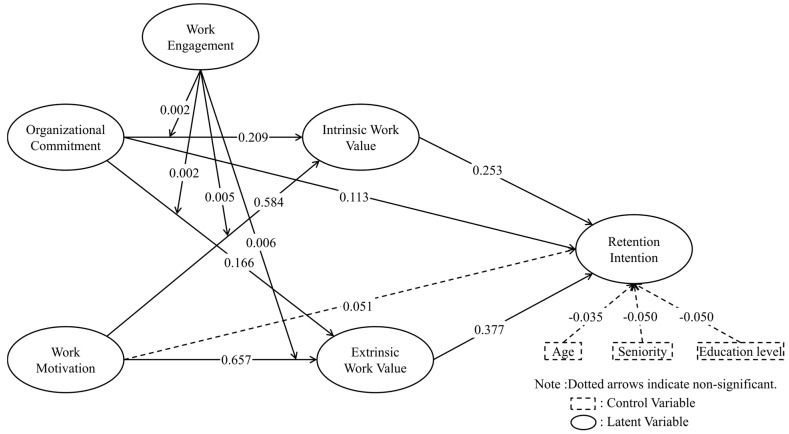
Results of Theoretical Framework. Note. The values are standardized regression coefficients. Dotted lines indicate insignificance.

As presented in Table 4 and Figure 2, the effect of organizational commitment on retention intention was significant (β = 0.113, *p* = 0.038); thus, H1 was supported. On the other hand, work motivation had no significant effect on retention intention (β = 0.051, *p* = 0.526), and H2 was not supported. However, organizational commitment showed a significant and positive effect on intrinsic work values (β = 0.209, *p* < 0.001), supporting H3, and also a significant positive effect on extrinsic work values (β = 0.166, *p* = 0.001), supporting H4. Work motivation had a significant positive effect on intrinsic work values (β = 0.584, *p* < 0.001), supporting H5, and on extrinsic work values (β = 0.657, *p* < 0.001), supporting H6. Regarding the effects of work values on retention intention, intrinsic work values significantly and positively influenced retention intention (β = 0.253, *p* < 0.001), supporting H7, while extrinsic work values also had a significant positive effect on retention intention (β = 0.377, *p* < 0.001), supporting H8.

To examine the mediating role of work values in the relationships among organizational commitment, Work Motivation, and retention intention, this study employed the bootstrapping method with 5000 resamples [61]. The results indicated that organizational commitment indirectly influenced retention intention through intrinsic work values (β = 0.053, *p* = 0.014), supporting H9, and through extrinsic work values (β = 0.063, *p* = 0.023), supporting H10. Similarly, work motivation indirectly influenced retention intention via intrinsic work values (β = 0.148, *p* = 0.006), supporting H11, and via extrinsic work values (β = 0.248, *p* < 0.001), supporting H12.

Regarding moderation effects, the results revealed that work engagement significantly moderated the relationship between organizational commitment and intrinsic work values (β = 0.002, *p* < 0.001), supporting H13, as well as the relationship between organizational commitment and extrinsic work values (β = 0.002, *p* < 0.001), supporting H14. Work engagement also significantly moderated the relationship between work motivation and intrinsic work values (β = 0.005, *p* < 0.001), supporting H15, and the relationship between work motivation and extrinsic work values (β = 0.006, *p* < 0.001), supporting H16.

## 5. Discussion

### 5.1. Work Values as a Key Mediating Role

This study’s findings indicate that the impact of work motivation on retention intention is not directly significant but is instead transmitted by work values. In the LTC context, work values are the key mediator that transforms motivation and commitment into retention intention. Expectancy theory holds that behavioral intention will be impacted by expectancy, instrumentality, and valence, with valence assessing the importance of outcomes. Work values embody the expressed valence form [15]. Employees review the reward examination based on perceived work values, which guides their retention choices [53].

Specifically, work values in this study correspond to the valence dimension of Expectancy Theory. Valence represents an individual’s subjective evaluation of the value and attractiveness of work outcomes [81], that is, the psychological intensity of motivation generated when employees perceive the valuable rewards that their efforts may bring. When LTC workers believe that their work fulfills intrinsic values such as helping others and achieving professional accomplishment, or extrinsic values such as stable pay and career security [53], these positive value perceptions constitute a high-valence psychological evaluation, thereby enhancing their commitment and intention to remain. In other words, work values embody employees’ cognitive judgments of work outcomes and rewards, representing the practical manifestation of the valence dimension in Expectancy Theory [22], which explains how value-based cognitive mechanisms transform into behavioral intentions.

Work values should not be static; they are fluid and adjusted dynamically based on work environments, institutional arrangements, and economic conditions [55]. In the LTC industry, where labor intensity is high, emotional labor is considerable, and workforce shortages exist, organizations could reinforce positive evaluations and retention tendencies when they provide hindsight and resources about employees’ values [59]. From a sustainability perspective, the stability of the LTC system relies on professionals who value their value. When organizations see sustainability as core to the organization and their work values are enhanced, it can help minimize the adverse effects of turnover on service quality, as high-quality care can continue, producing sustainable development in the LTC system.

### 5.2. Comparison of Intrinsic and Extrinsic Values

The empirical results of this study show that in the LTC context, extrinsic values exert a more substantial effect; however, intrinsic and extrinsic work values function complementarily in shaping retention intention and are closely tied to sustainable human resource development. According to expectancy theory, motivation derives from the interaction of expectancy, instrumentality, and valence, with work values representing the core source of valence [15]. Intrinsic values emphasize task meaning, self-fulfillment, and challenge [53], while extrinsic values focus on working conditions such as salary, stability, benefits, and flexibility [55].

The findings suggest that the two types of values are not mutually exclusive; employees may value both simultaneously, but the psychological connections differ: intrinsic and social values are often associated with need satisfaction, whereas extrinsic and status values are more likely linked to need frustration [56]. Empirical evidence indicates that both positively influence retention intention, with extrinsic values exerting a greater impact, reflecting that the instrumentality and visibility of external rewards are more easily perceived and assessed by employees. Moreover, work motivation enhances intrinsic and extrinsic values, while organizational commitment primarily promotes intrinsic values, clarifying the connection between effort and reward.

In addition, during periods of economic instability or increased employment risks, employees place greater emphasis on tangible external rewards [60]. Therefore, LTC institutions should establish fair and predictable compensation and benefit mechanisms while simultaneously materializing intrinsic rewards through empowerment, professional development, and learning opportunities. Such measures can enhance expectancy, instrumentality, and valence, thereby strengthening retention intention, ensuring workforce stability, and sustaining service quality to support the long-term development goals of the LTC system.

### 5.3. Work Engagement as a “Signal Amplifier”

This study found that work engagement strengthens the effects of organizational commitment and work motivation on work values among LTC employees. Work engagement is a positive psychological state comprising vigor, dedication, and absorption [60], and functions as a signal amplifier that enhances the process through which commitment and motivation are transformed into values. Organizational commitment involves identifying organizational goals, willingness to make sustained contributions, and an intention to remain, encompassing affective, normative, and continuance dimensions. When employees exhibit high work engagement, positive signals associated with commitment—such as goal alignment and feedback availability—become more salient, reinforcing the linkage between effort, performance, and outcomes. Within the framework of expectancy theory, this amplification effect enhances employees’ evaluation of expectancy and instrumentality, making it easier for commitment to be converted into work values, which themselves represent standards for assessing outcome desirability and are closely tied to individual beliefs and preferences [54].

Work motivation provides direction, intensity, and persistence of effort, and is consistently associated with performance [36]. Under conditions of high work engagement, its positive influence on work values becomes more pronounced. The enthusiasm and sense of meaning that accompany high engagement heighten employees’ sensitivity to achievement and growth rewards, while reducing the likelihood of experiencing value frustration in pursuing goals [68]. Thus, motivation-driven efforts are more readily recognized as yielding valued outcomes, enhancing overall work values.

In the context of sustainable development in the LTC industry, the amplifying role of work engagement holds dual significance. On the one hand, it increases the effectiveness of translating commitment and motivation into values, stabilizing employees’ retention intentions. On the other hand, it contributes to long-term workforce stability and sustained service quality, reducing care interruptions and cost inefficiencies caused by turnover. When organizations strategically emphasize commitment, motivation, and engagement simultaneously, a positive cycle of expectancy, instrumentality, and valence can be established to support the sustainability goals of the LTC service system.

### 5.4. Theoretical Implications

To strengthen employees’ work engagement and retention stability in LTC institutions, managers should establish effective social exchange relationships and, drawing on the perspective of expectancy theory, clearly link the relationships among effort, performance, and rewards. When employees perceive that their efforts can lead to tangible performance and that such performance can be converted into valued rewards, they are more likely to sustain motivation and engagement. These rewards include extrinsic values such as salary, benefits, and career development and intrinsic values such as achievement, recognition, and work meaningfulness, enhancing retention intention and care quality. Within workforce shortages and high emotional demands in LTC institutions [82], work engagement indicates psychological state and is a key mechanism connecting valence in expectancy theory. This study suggests that managers should simultaneously strengthen organizational commitment and value-response mechanisms, ensuring that employees experience extrinsic rewards (e.g., promotion, incentives, job security) and intrinsic fulfillment (e.g., professional accomplishment, altruistic value, emotional connection) when exerting effort.

### 5.5. Practical Implications

The findings of this study provide important implications for the management of LTC institutions. First, work engagement is critical to retention intention and service quality. Institutions may adopt high performance work systems to enhance employees’ skills and psychological engagement through professional training and emotional support, enabling caregivers to maintain stable, high-quality services despite high pressure and emotional labor [83]. Second, fulfilling caregivers’ needs for work values significantly influences their motivation and retention. When extrinsic values such as salary, benefits, and career development, and intrinsic values such as achievement, meaningfulness of helping others, and emotional connection are satisfied, employees are more likely to sustain long-term commitment and stable service [30]. Therefore, institutions should establish comprehensive reward and recognition systems that allow employees to feel valued daily, for example, establishing an “ethical recognition culture” through initiatives such as “caring story sharing” or an “ethical performance award” can make employees’ helping behaviors and emotional contributions more visible and appreciated. Enhancing their retention intention. Finally, LTC institutions should provide a clear role and performance expectations. In line with expectancy theory, employees’ willingness to engage and perform improves when they understand the relationship between effort and performance and trust that performance will lead to valued outcomes. However, performance should not be limited to quantifiable service efficiency but should also encompass the preservation of elders’ dignity, privacy, and the value of life. Therefore, managers should provide regular ethical feedback, career counseling, and communication with care attendants to help them maintain high work engagement under clear guidance and predictable rewards, while upholding the ethical essence of caregiving. This approach can simultaneously promote workforce stability, enhance service quality, and strengthen the humanistic and ethical value foundation of LTC institutions.

### 5.6. Limitations and Future Research

The research is based on a single industry and sample source, which limits the study’s external validity. Nevertheless, the study has made both practical and theoretical contributions in examining the antecedents of retention intention, particularly the link between organizational commitment and work values. While the sample and the data did not elicit the rich characteristics of personnel and organizational contexts from different LTC institutions, future research could potentially use multilevel designs that capture both employee- and organization-level data to examine better the direct and/or indirect effects of management strategies, organizational culture, and resource allocations upon retention intention to overcome the limitations of a single level analysis. Furthermore, future research could consider examining different regions or types of LTC institutions to understand better the applicability of the current findings across organizational contexts and to examine cross-cultural comparisons to understand better if culture influences the mechanisms that inform retention intention. By conducting multilevel and multi-context analyses and adding cross-cultural comparisons, a richer and more generalized theoretical model could be developed, coupled with a greater evidence base for retention strategies across the LTC sector.

## 6. Conclusions

This study examined employees in LTC institutions to verify the mediating role of work values in the process by which organizational commitment and work motivation influence retention intention, interpreted through the lens of expectancy theory. The results indicate that employees’ retention intention is more stable when they perceive that effort leads to performance, performance translates into tangible rewards, and such rewards align with valued intrinsic and extrinsic work values. The findings further reveal the complementary nature of intrinsic and extrinsic values, with extrinsic values being more influential in the LTC context. In addition, work engagement demonstrates an “amplifier” effect by strengthening the transformation of commitment and motivation into value perceptions. Theoretically, this study extends the application of expectancy theory to human resource management in LTC services. Practically, it suggests that LTC institutions should adopt high-performance work systems, fair reward mechanisms, and emotional support to meet both intrinsic and extrinsic values, thereby enhancing employee engagement and retention intention.

## Figures and Tables

**Table 1:** Distribution of Demographic Characteristics.

Category		Frequency	Percentage (%)
Gender	Male	108	21.6
	Female	393	78.4
Age	21–30 years	30	6.0
	31–40 years	30	6.0
	41–50 years	94	18.8
	51–60 years	160	31.9
	Above 61 years	187	37.3
Seniority	Within 6 months	39	7.8
	6 months to 1 year	24	4.8
	1 year to 2 years	57	11.4
	2 years to 5 years	132	26.3
	5 years to 15 years	180	35.9
	15 years to 20 years	43	8.6
	More than 20 years	26	5.2
Education level	Junior high school or below	147	29.3
	Senior/vocational high school	243	48.5
	College	59	11.8
	Graduate	52	10.4

**Table 2:** Confirmatory Factor Analysis and Scale Reliability.

Items	Unstd.	S.E.	t	*p*	Std.	α	CR	AVE
Organizational Commitment (OC)						0.929	0.931	0.599
OC1	1				0.789			
OC2	1.002	0.054	18.491	<0.001	0.758			
OC3	1.084	0.056	19.471	<0.001	0.789			
OC4	1.134	0.059	19.108	<0.001	0.778			
OC5	1.070	0.063	16.912	<0.001	0.706			
OC6	1.129	0.056	20.281	<0.001	0.814			
OC7	1.108	0.057	19.435	<0.001	0.788			
OC8	0.975	0.053	18.555	<0.001	0.760			
OC9	1.239	0.065	19.035	<0.001	0.775			
Work Motivation (WM)						0.919	0.923	0.547
WM1	1				0.800			
WM2	0.958	0.047	20.547	<0.001	0.810			
WM3	0.954	0.051	18.696	<0.001	0.755			
WM4	0.960	0.051	18.731	<0.001	0.756			
WM5	0.859	0.047	18.265	<0.001	0.741			
WM6	0.880	0.044	19.830	<0.001	0.789			
WM7	0.974	0.055	17.720	<0.001	0.724			
WM8	0.956	0.052	18.361	<0.001	0.744			
WM9	1.046	0.067	15.510	<0.001	0.650			
WM10	1.030	0.073	14.141	<0.001	0.602			
Intrinsic Work Values (IWV)						0.885	0.887	0.612
IWV1	1				0.842			
IWV2	0.873	0.045	19.422	<0.001	0.764			
IWV3	0.987	0.049	20.074	<0.001	0.782			
IWV4	0.921	0.047	19.763	<0.001	0.773			
IWV5	0.913	0.049	18.816	<0.001	0.746			
Extrinsic Work Values (EWV)						0.859	0.861	0.554
EWV1	1				0.744			
EWV2	0.963	0.056	17.189	<0.001	0.789			
EWV3	1.070	0.067	16.030	<0.001	0.737			
EWV4	1.022	0.066	15.460	<0.001	0.712			
EWV5	0.926	0.058	15.989	<0.001	0.736			
Retention Intention(RI)						0.945	0.945	0.712
RI1	1				0.852			
RI2	0.889	0.036	25.009	<0.001	0.856			
RI3	0.970	0.045	21.621	<0.001	0.785			
RI4	0.919	0.043	21.448	<0.001	0.781			
RI5	0.900	0.034	26.548	<0.001	0.884			
RI6	0.866	0.034	25.618	<0.001	0.867			
RI7	0.954	0.037	25.975	<0.001	0.874			
Work Engagement (WE)						0.902	0.905	0.514
WE1	1				0.695			
WE2	1.078	0.071	15.168	<0.001	0.735			
WE3	1.013	0.069	14.699	<0.001	0.711			
WE4	0.815	0.062	13.189	<0.001	0.634			
WE5	1.429	0.092	15.602	<0.001	0.758			
WE6	1.112	0.072	15.394	<0.001	0.747			
WE7	1.158	0.076	15.179	<0.001	0.736			
WE8	0.899	0.057	15.753	<0.001	0.766			
WE9	0.807	0.059	13.733	<0.001	0.661			

Note: The abbreviations in the table are defined as follows: Unstd.: Unstandardized Estimate; S.E.: Standard Error; t: t-value; *p*: *p*-value; Std.: Standardized Estimate; α: Cronbach’s Alpha; CR: Composite Reliability; AVE: Average Variance Extracted.

**Table 3:** Discriminant Validity Assessment.

Constructs	Mean	SD	AVE	Discriminant Validity					
				OC	WM	IWV	EWV	RI	WE
Organizational Commitment (OC)	3.881	0.657	0.599	**0.774**					
Work Motivation (WM)	4.230	0.544	0.547	0.629	**0.740**				
Intrinsic Work Values (IWV)	4.386	0.487	0.612	0.541	0.615	**0.782**			
Extrinsic Work Values (EWV)	4.346	0.518	0.554	0.524	0.635	0.874	**0.744**		
Retention Intention (RI)	4.279	0.782	0.712	0.471	0.521	0.607	0.623	**0.844**	
Work Engagement (WE)	4.277	0.548	0.514	0.140	0.216	0.185	0.200	0.096	**0.717**

Note: AVE stands for average variance extracted. The numbers in the lower triangle of the discriminant validity matrix represent Pearson correlation coefficients, while the numbers on the diagonal represent the square root of AVE. The abbreviations in the table are defined as follows: SD.: Standard Deviation; AVE: Average Variance Extracted.

**Table 4:** Path Coefficients and Significances.

Path Analysis	Std.	Unstd.	S.E.	*p*
Control Variables				
Age→Retention Intention	−0.035	−0.020	0.023	0.383
Seniority→Retention Intention	−0.050	−0.027	0.021	0.181
Education level→Retention Intention	−0.050	−0.044	0.033	0.185
Hypothesis				
H1: Organizational Commitment→Retention Intention	0.113	0.155	0.075	0.038
H2:Work Motivation→Retention Intention	0.051	0.077	0.121	0.526
H3: Organizational Commitment→Intrinsic Work Values	0.209	0.174	0.043	<0.001
H4: Organizational Commitment→Extrinsic Work Values	0.166	0.137	0.042	0.001
H5: Work Motivation→Intrinsic Work Values	0.584	0.533	0.051	<0.001
H6: Work Motivation→Extrinsic Work Values	0.657	0.589	0.054	<0.001
H7: Intrinsic Work Values→Retention Intention	0.253	0.413	0.099	<0.001
H8: Extrinsic Work Values→Retention Intention	0.377	0.627	0.117	<0.001
H9: Organizational Commitment→Intrinsic Work Values→Retention Intention	0.053	0.072	0.041	0.014
H10: Organizational Commitment→Extrinsic Work Values→Retention Intention	0.063	0.086	0.049	0.023
H11: Work Motivation→Intrinsic Work Values→Retention Intention	0.148	0.220	0.101	0.006
H12: Work Motivation→Extrinsic Work Values→Retention Intention	0.248	0.369	0.129	<0.001
H13: Work Engagement*Organizational Commitment (moderation effect)→Intrinsic Work Values	0.002	0.009	0.001	<0.001
H14: Work Engagement*Organizational Commitment (moderation effect)→Extrinsic Work Values	0.002	0.010	0.001	<0.001
H15: Work Engagement*Work Motivation (moderation effect)→Intrinsic Work Values	0.005	0.009	0.001	<0.001
H16: Work Engagement*Work Motivation (moderation effect)→Extrinsic Work Values	0.006	0.012	0.001	<0.001

Note: Bootstrapping = 5000. The abbreviations in the table are defined as follows: Std.: Standardized Estimate; Unstd.: Unstandardized Estimate; S.E.: Standard Error; *p*: *p*-value.

## Data Availability

The data presented in this study are available on request from the corresponding author due to privacy restrictions concerning employees’ work conditions.

## Appendix A

**Table A1:** Questionnaire Items Used for LTC Employees.

Item	Description	Source
Organizational Commitment		[67]
OC1	I hope to continue developing my career in the current long-term care institution.	
OC2	I regard the problems faced by the current long-term care institution as my own problems.	
OC3	I feel like a member of a family in the current long-term care institution.	
OC4	The current long-term care institution provides me with a great deal of support in my life.	
OC5	I believe that my contribution to the current long-term care institution is still limited.	
OC6	I feel that the current long-term care institution is worthy of my long-term loyalty and service.	
OC7	I find it difficult to leave the current long-term care institution because I am concerned that I may not be able to find a job elsewhere.	
OC8	Leaving the current long-term care institution may have negative consequences for me.	
OC9	It is not easy to find another long-term care job with stable income like the current one.	
Work motivation		[68]
WM1	The tasks I perform at work themselves drive my motivation to work.	
WM2	The tasks I perform at work are enjoyable.	
WM3	My work is meaningful.	
WM4	My work is very exciting.	
WM5	My work is so interesting that it is a motivation in itself.	
WM6	Sometimes I feel so inspired by my work that I almost forget everything around me.	
WM7	If I need to put in extra effort at work, I need to receive extra compensation.	
WM8	For me, it is important to have external reward goals in order to be motivated to do my work well.	
WM9	External rewards such as bonuses and allowances are necessary for my work performance.	
WM10	If I could receive better pay, I would perform better.	
Intrinsic work values		[69]
IWV1	This long-term care job provides opportunities to exercise initiative.	
IWV2	This long-term care job provides me with a sense of accomplishment.	
IWV3	This long-term care job provides me with a sense of responsibility.	
IWV4	This long-term care job matches my abilities.	
IWV5	This long-term care job is interesting.	
Extrinsic work values		[69]
EWV1	This long-term care job provides generous vacation.	
EWV2	This long-term care job offers good working hours.	
EWV3	This long-term care job does not involve excessive stress.	
EWV4	This long-term care job provides good salary.	
EWV5	This long-term care job offers good job security.	
Retention Intention		[70]
RI1	I have hardly ever thought about leaving this long-term care institution.	
RI2	I feel that staying in this long-term care institution is the right choice.	
RI3	Even if there were better job opportunities, I would not want to leave this long-term care institution.	
RI4	I feel a responsibility to continue working in this long-term care institution.	
RI5	I would feel guilty if I were to leave this long-term care institution.	
RI6	No matter how this long-term care institution changes, I do not want to leave.	
RI7	I am very loyal to this long-term care institution.	
Work Engagement		[71]
WE1	When I am working, I feel full of energy.	
WE2	In my job, I feel strong and vigorous.	
WE3	I am enthusiastic about my work.	
WE4	My job inspires me.	
WE5	When I wake up in the morning, I feel like going to work.	
WE6	I feel happy when I am working intensely.	
WE7	I am proud of the work that I do.	
WE8	I am fully concentrated on my work.	
WE9	I get carried away when I am working.	

## References

[1] Teng J.-K., Liu H.-L. Elder Care as a Focus of Social Concern: The Multifunctional Long-Term Care and Wellness Village as a New Trend. https://www.ydn.com.tw/news/newsInsidePage?chapterID=1735819.

[2] Tai C.-Y. Embracing a New Future in 2025: Outlook on Nine Key Trends in the Care Industry Review and Prospects. https://www.ankecare.com/article/3483-2025-01-12-16-21-15.

[3] Tazi Y.C., Yang H.Y., Lai F.T. (2019). A study on turnover factor of nurse aides. J. Perform. Strategy Res..

[4] Liu Q., Zhao H. (2023). From committed employees to rebels: The role of prosocial rule-breaking, age, and entrepreneurial self-efficacy. Int. J. Hum. Resour. Manag..

[5] Holtom B.C., Burch T.C. (2016). A model of turnover-based disruption in customer services. Hum. Resour. Manag. Rev..

[6] Porter C.M., Rigby J.R. (2021). The turnover contagion process: An integrative review of theoretical and empirical research. J. Organ. Behav..

[7] Azmy A., Perkasa D.H., Haryadi A., Priyono A. (2025). The roles of competence and job satisfaction on sales insurance performance: Organizational commitment as mediating variable. Qual.-Access Success.

[8] Pinder C.C. (2014). Work Motivation in Organizational Behavior.

[9] Kotera Y., Van Laethem M., Ohshima R. (2020). Cross-cultural comparison of mental health between Japanese and Dutch workers: Relationships with mental health shame, self-compassion, work engagement and motivation. Cross Cult. Strateg. Manag..

[10] Deci E.L., Olafsen A.H., Ryan R.M. (2017). Self-determination theory in work organizations: The state of a science. Annu. Rev. Organ. Psychol. Organ. Behav..

[11] Wang N., Luan Y., Ma R. (2024). Detecting causal relationships between work motivation and job performance: A meta-analytic review of cross-lagged studies. Humanit. Soc. Sci. Commun..

[12] Csikszentmihalyi M., Seligman M. (2000). Positive psychology. Am. Psychol..

[13] Vroom H.V. (1964). Work and Motivation.

[14] Gyepi-Garbrah T., Preko A., Mohammed I., Mohammed I. (2023). Using goal-setting theory and expectancy theory to understand career goal implementation in the hospitality industry. J. Hosp. Leis. Sport Tour. Educ..

[15] Osafo E., Paros A., Yawson R.M. (2021). Valence–instrumentality–expectancy model of motivation as an alternative model for examining ethical leadership behaviors. Sage Open.

[16] Aboramadan M., Hassi A., Alharazin H.J., Dahleez K.A., Albashiti B. (2019). Volunteering drivers and continuation will: The role of engagement. J. Manag. Dev..

[17] Karatepe O.M., Olugbade O.A. (2016). The mediating role of work engagement in the relationship between high-performance work practices and job outcomes of employees in Nigeria. Int. J. Contemp. Hosp. Manag..

[18] Aboramadan M. (2022). The effect of green HRM on employee green behaviors in higher education: The mediating mechanism of green work engagement. Int. J. Organ. Anal..

[19] Ranay F. (2025). Examining the interplay dynamics of innovative work environment, work motivation and organizational commitment. Div. Word Int. J. Manag. Humanit..

[20] Yusnita N. (2022). Investigating work value, job satisfaction and organizational commitment among national electric company employees. J. Bisnis Manag..

[21] Fan A., Kline S.F., Liu Y., Byrd K. (2022). Consumers’ lodging intentions during a pandemic: Empirical insights for crisis management practices based on protection motivation theory and expectancy theory. Int. J. Contemp. Hosp. Manag..

[22] Zhang Y., Zhou X., Zhang H., Khanagha S. (2025). Mechanisms of vision communication and employees’ change-supportive behavior from the perspective of expectancy theory. J. Bus. Res..

[23] Deci E.L., Ryan R.M. (1985). The general causality orientations scale: Self-determination in personality. J. Res. Pers..

[24] Ryan R.M., Deci E.L. (2000). Intrinsic and extrinsic motivations: Classic definitions and new directions. Contemp. Educ. Psychol..

[25] Nguyen T.V.T., Ryan R.M., Deci E.L. (2018). Solitude as an approach to affective self-regulation. Pers. Soc. Psychol. Bull..

[26] Green-Demers I., Legault L., Pelletier D., Pelletier L.G. (2008). Factorial invariance of the Academic Amotivation Inventory (AAI) across gender and grade in a sample of Canadian high school students. Educ. Psychol. Meas..

[27] Redondo R., Sparrow P., Hernández-Lechuga G. (2021). The effect of protean careers on talent retention: Examining the relationship between protean career orientation, organizational commitment, job satisfaction and intention to quit for talented workers. Int. J. Hum. Resour. Manag..

[28] Wieschollek V., Dlouhy K. (2023). Employee referrals as counterproductive work behavior? Employees’ motives for poor referrals and the role of the cultural context. Int. J. Hum. Resour. Manag..

[29] Marcoux G., Guihur I., Leclerc A. (2021). Co-operative difference and organizational commitment: The filter of socio-demographic variables. Int. J. Hum. Resour. Manag..

[30] Kim M., Beehr T.A. (2020). Empowering leadership: Leading people to be present through affective organizational commitment?. Int. J. Hum. Resour. Manag..

[31] Redondo R., Sparrow P., Hernández-Lechuga G. (2023). Proactive career orientation and physical mobility preference as predictors of important work attitudes: The moderating role of pay satisfaction. Int. J. Hum. Resour. Manag..

[32] Ausar K., Kang H.J.A., Kim J.S. (2016). The effects of authentic leadership and organizational commitment on turnover intention. Leadersh. Organ. Dev. J..

[33] Ramalho Luz C.M.D., Luiz de Paula S., de Oliveira L.M.B. (2018). Organizational commitment, job satisfaction and their possible influences on intent to turnover. Rev. Gestão.

[34] Jun W.H. (2025). The mediating effects of nurses’ professional values on the relationship between work environment and organizational commitment among long-term care hospital nurses. BMC Nurs..

[35] Saito Y., Igarashi A., Noguchi-Watanabe M., Takai Y., Yamamoto-Mitani N. (2018). Work values and their association with burnout/work engagement among nurses in long-term care hospitals. J. Nurs. Manag..

[36] Li A., Zhou Z.E., Shao P.T., Lin Q. (2023). The father’s and the mother’s intrinsic work motivation and their work-to-family conflict perceived by the adolescent: Dyadic and triadic analyses. J. Organ. Behav..

[37] Van den Broeck A., Ferris D.L., Chang C.H., Rosen C.C. (2016). A review of self-determination theory’s basic psychological needs at work. J. Manag..

[38] Jang M., Aavakare M., Nikou S., Kim S. (2021). The impact of literacy on intention to use digital technology for learning: A comparative study of Korea and Finland. Telecommun. Policy.

[39] Zhao L., Cao C., Li Y., Li Y. (2022). Determinants of the digital outcome divide in e-learning between rural and urban students: Empirical evidence from the COVID-19 pandemic based on capital theory. Comput. Hum. Behav..

[40] Morkevičiūtė M., Endriulaitienė A. (2020). Explaining work motivation through perceived transformational leadership: What to expect in a sample of female employees?. Gend. Manag. Int. J..

[41] Saleh M.O., Eshah N.F., Rayan A.H. (2022). Empowerment predicting nurses’ work motivation and occupational mental health. SAGE Open Nurs..

[42] Trépanier S.G., Vallerand R.J., Ménard J., Peterson C. (2020). Job resources and burnout: Work motivation as a moderator. Stress Health.

[43] HakemZadeh F., Chowhan J., Neiterman E., Zeytinoglu I., Geraci J., Lobb D. (2024). Differential relationships between work-life interface constructs and intention to stay in or leave the profession: Evidence from midwives in Canada. Psychol. Rep..

[44] Milliman J., Gatling A., Kim J.S. (2018). The effect of workplace spirituality on hospitality employee engagement, intention to stay, and service delivery. J. Hosp. Tour. Manag..

[45] Kung S., Ibrahimi A., Biru A. (2024). Examining the mediating role of perceived organizational support in the relationship between employee motivation and intention to stay: A study of millennials in Malaysia. Manag. J. Contemp. Manag. Issues.

[46] Mardanov I. (2021). Intrinsic and extrinsic motivation, organizational context, employee contentment, job satisfaction, performance and intention to stay. Evid.-Based HRM.

[47] Rubenstein A.L., Eberly M.B., Lee T.W., Mitchell T.R. (2018). Surveying the forest: A meta-analysis, moderator investigation, and future-oriented discussion of the antecedents of voluntary employee turnover. Pers. Psychol..

[48] Valéau P., Paille P., Dubrulle C., Guenin H. (2021). The mediating effects of professional and organizational commitment on the relationship between HRM practices and professional employees’ intention to stay. Int. J. Hum. Resour. Manag..

[49] Husin W.N.W., Kernain N.F.Z. (2020). The influence of individual behaviour and organizational commitment towards the enhancement of Islamic work ethics at Royal Malaysian Air Force. J. Bus. Ethics.

[50] Birtch T.A., Cai Z., Chiang F.F. (2024). Effects of formal mentoring support on newcomer–protégé affective organizational commitment: A self-concept-based perspective. Hum. Resour. Manag..

[51] Dousin O., Wei C.X., Balakrishnan B.K., Lee M.C.C. (2021). Exploring the mediating role of flexible working hours in the relationship of supervisor support, job and life satisfaction: A study of female nurses in China. Nurs. Open.

[52] Rokeach M. (1973). The Nature of Human Values.

[53] Ede M.O., Okeke C.I., Adene F., Areji A.C. (2023). Perceptions of work value and ethical practices amongst primary school teachers, demographics, intervention, and impact. Psychol. Rep..

[54] Song Y., Gao S., Zhao Y., Singh Gaur S. (2020). What do we still need to know about employee creativity: A fsQCA approach. Sustainability.

[55] Cao Y. (2020). Economic development, market transition, and work values in post-socialist China. Soc. Forces.

[56] Busque-Carrier M., Ratelle C.F., Le Corff Y. (2022). Linking work values profiles to basic psychological need satisfaction and frustration. Psychol. Rep..

[57] Li G., Zhang G., Zhang X., Martek I., Chen D. (2025). The formation mechanism of employees’ turnover intention in AEC industry. Buildings.

[58] Miller-Mor-Attias R., Vigoda-Gadot E. (2022). Evolving motivation in public service: A three-phase longitudinal examination of public service motivation, work values, and academic studies among Israeli students. Public Adm. Rev..

[59] Lu L., Cooper C.L. (2022). Sickness presenteeism as a link between long working hours and employees’ outcomes: Intrinsic and extrinsic motivators as resources. Int. J. Environ. Res. Public Health.

[60] Vranken I., Vandenbosch L. (2024). Social and vocational identity in workers’ online posts: A large-scale Instagram content analysis of job-related hashtags. Behav. Inf. Technol..

[61] Memon M.A., Salleh R., Mirza M.Z., Cheah J.H., Ting H., Ahmad M.S., Tariq A. (2021). Satisfaction matters: The relationships between HRM practices, work engagement and turnover intention. Int. J. Manpow..

[62] Kahn W.A. (1990). Psychological conditions of personal engagement and disengagement at work. Acad. Manag. J..

[63] Aldabbas H., Pinnington A., Lahrech A. (2023). The influence of perceived organizational support on employee creativity: The mediating role of work engagement. Curr. Psychol..

[64] Uka A., Prendi A. (2021). Motivation as an indicator of performance and productivity from the perspective of employees. Manag. Mark..

[65] Hashiguchi N., Sengoku S., Kubota Y., Kitahara S., Lim Y., Kodama K. (2021). Age-dependent influence of intrinsic and extrinsic motivations on construction worker performance. Int. J. Environ. Res. Public Health.

[66] Brislin R.W. (1970). Back-translation for cross-cultural research. J. Cross-Cult. Psychol..

[67] Meyer J.P., Stanley D.J., Herscovitch L., Topolnytsky L. (2002). Affective, continuance, and normative commitment to the organization: A meta-analysis of antecedents, correlates, and consequences. J. Vocat. Behav..

[68] Kuvaas B., Buch R., Weibel A., Dysvik A., Nerstad C.G. (2017). Do intrinsic and extrinsic motivation relate differently to employee outcomes?. J. Econ. Psychol..

[69] Liang Y.W. (2012). The relationships among work values, burnout, and organizational citizenship behaviors: A study from hotel front-line service employees in Taiwan. Int. J. Contemp. Hosp. Manag..

[70] Chen C.L., Chen M.H. (2021). Hospitality industry employees’ intention to stay in their job after the COVID-19 pandemic. Adm. Sci..

[71] Labrague L.J., Obeidat A.A. (2022). Transformational leadership as a mediator between work–family conflict, nurse-reported patient safety outcomes, and job engagement. J. Nurs. Scholarsh..

[72] Hayes A.F. (2009). Beyond Baron and Kenny: Statistical mediation analysis in the new millennium. Commun. Monogr..

[73] Ping R.A. (1995). A parsimonious estimating technique for interaction and quadratic latent variables. J. Mark. Res..

[74] Williams L.J., Brown B.K. (1994). Method variance in organizational behavior and human resources research: Effects on correlations, path coefficients, and hypothesis testing. Organ. Behav. Hum. Decis. Process..

[75] Podsakoff P.M., MacKenzie S.B., Lee J.Y., Podsakoff N.P. (2003). Common method biases in behavioral research: A critical review of the literature and recommended remedies. J. Appl. Psychol..

[76] Peng T.K., Kao Y.T., Lin C.C. (2006). Common method variance in management research: Its nature, effects, detection, and remedies. J. Manag..

[77] Kline R.B. (2023). Principles and Practice of Structural Equation Modeling.

[78] Hair J.F., Black W.C., Babin B.J., Anderson R.E., Tatham R.L. (2017). Multivariate Data Analysis.

[79] Bagozzi R.P., Yi Y. (2012). Specification, evaluation, and interpretation of structural equation models. J. Acad. Mark. Sci..

[80] West S.G., Taylor A.B., Wu W., Hoyle R.H. (2012). Model fit and model selection in structural equation modeling. Handbook of Structural Equation Modeling.

[81] Dieser K.H. (2025). Applicability of the VIE-model in public administration. Eur. J. Psychol. Res..

[82] Shepherd-Banigan M., Jones K.A., Wang K., DePasquale N., Van Houtven C., Olsen J.M. (2020). Mechanisms through which a family caregiver coaching intervention might reduce anxiety among children in military households. Matern. Child Health J..

[83] Han J.H., Kang S., Oh I.S., Kehoe R.R., Lepak D.P. (2019). The Goldilocks effect of strategic human resource management? Optimizing the benefits of a high-performance work system through the dual alignment of vertical and horizontal fit. Acad. Manag. J..

